# Proceedings of the inaugural Canadian Healthcare Navigation Conference: a forum for sharing innovations and best practices in navigation services

**DOI:** 10.1186/s12919-021-00229-0

**Published:** 2021-11-30

**Authors:** R. Markoulakis, A. Luke, A. Reid, K. Mehra, A. Levitt, S. Doucet

**Affiliations:** 1 Family Navigation Project, Sunnybrook Research Institute, Toronto, Canada; 2 Department of Occupational Science and Occupational Therapy, Faculty of Medicine, University of Toronto, Toronto, Canada; 3 Department of Nursing and Health Sciences, University of New Brunswick Saint John, Saint John, Canada; 4 Centre for Research in Integrated Care, University of New Brunswick, St. John, Canada; 5 Department of Psychiatry, Faculty of Medicine, University of Toronto, Toronto, Canada; 6 Hurvitz Brain Sciences Program, Sunnybrook Health Sciences Centre, Toronto, Canada

## Abstract

**Background:**

Individuals experiencing chronic illnesses face many physical, emotional, and social strains as a result of their illnesses, all the while trying to navigate unfamiliar territory in the healthcare system. Navigation is a strategy that can help people facing complex care needs and barriers to care in finding and accessing needed supports in the health care system. Navigators provide a patient-centred service, guiding individuals through their care plans and overcoming barriers to care. Navigation supports for individuals experiencing complex care needs have shown significant promise and have been gaining traction across Canada.

**Methods:**

The Canadian Healthcare Navigation Conference was the first event of its kind in Canada to bring together navigation researchers, service providers, students, decision makers, and individuals with lived experience to share lessons learned, promising practices, and research findings. This event was co-hosted by the Family Navigation Project at Sunnybrook Health Sciences Centre and NaviCare/SoinsNavi at the University of New Brunswick, and took place virtually on April 15–16, 2021.

**Results:**

This event spanned two days, which both began with a keynote address, one from a researcher and medical professional in navigation, and another from an individual with lived experience involved in advocacy in Canadian healthcare. Concurrent oral presentations by a variety of presenters were held following each keynote presentation. A poster session was held at the end of the first day, and a panel presentation rounded out the second day. Concurrent and poster presentations covered a range of topics pertaining to approaches to navigation, navigator roles, evaluation and quality improvement, lived experience in navigation, and navigation in the context of the COVID-19 pandemic. The panel presentation focused on identifying how the navigation field has progressed in Canada and identifying crucial next steps in navigation. These next steps were determined to be: 1) agreement on navigation-related definitions, 2) regulation and training, 3) equity, diversity, inclusion, and accessibility, 4) integrating lived experience, and 5) regional coordination.

**Conclusion:**

This conference was an important first step to creating a shared national conversation about navigation services so that we can continue to develop, implement, and share best evidence and practices in the field.

## Navigation services in healthcare: the Canadian context

People experiencing chronic illnesses - such as cancer, HIV, mental health or addictions issues, and dementia - are often faced with the physical, emotional, and social strains of their illnesses, all the while trying to navigate unfamiliar territory in the healthcare system. Patient navigation (referred to hereafter as navigation), was developed as a clinical service to help clients address care disparities that arise as a result of complex and fragmented care systems fraught with financial, communication, information, systemic, and personal barriers [1]. Navigators provide a client-centred service, guiding individuals through their care and eliminating barriers to timely access to support [1]. Since its first introduction in breast cancer care, navigation has been applied to diverse populations with chronic illnesses, such as other cancers and HIV [2, 3] where evidence suggests navigation can improve screening rates as well as access and adherence to treatment [4]. Navigation reduces barriers; connects clients to appropriate resources and supports in a timely manner; and empowers clients in managing their health [5–9]. While navigation programs have shown significant promise and recently gained traction across Canada [10, 11], the majority of evidence to date has emerged from the United States [12]. A forum was needed for Canadian healthcare navigation researchers, service providers, decision makers, individuals with lived experience, and other relevant stakeholders to come together and share lessons learned, promising practices, and research findings. The inaugural *Canadian Healthcare Navigation Conference*, co-hosted by the Family Navigation Project at Sunnybrook Health Sciences Centre and NaviCare/SoinsNavi at the University of New Brunswick in Saint John, took place virtually on April 15 and 16, 2021, to facilitate and stimulate this important national conversation.

Frontline [13], academic [10], and policy [14] stakeholders have described navigation support as critical for patient care. Furthermore, navigation has been recognized as becoming the “norm” in Canadian healthcare [15]. The central premise of navigation is to proactively guide, support, and orient clients through healthcare systems, matching clients’ unmet needs to appropriate resources to decrease fragmentation, improve access, and promote integrated care [16]. Navigation services are committed to being timely, responsive, and accessible. Navigation can support whole families and, in many cases, indirectly support the recipients of treatment by working with family caregivers to find the most appropriate path. There are several navigation-type services in Canada that have emerged over the past several years serving a range of target populations and using diverse service delivery models. For example, promising practices in Canada include: British Columbia’s FamilySmart [17] and CMHA Family Navigator [18]; Ontario’s Children’s Hospital of Eastern Ontario Navigator [19], Pinecrest-Queensway System Navigation [20], Parents’ Lifeline of Eastern Ontario [21], FirstLink Alzheimer Society Navigation [22], and Family Navigation Project [9]; Newfoundland’s Provincial Mental Health and Addictions System Navigator [16]; and New Brunswick’s NaviCare/SoinsNavi [23] and Pediatric Oncology Navigator [24], to name a few.

Evidence regarding the value of navigation for care providers and the healthcare system is also mounting in Canada. For example, a pilot navigator program in British Columbia demonstrated improved communication between the many agencies involved in patient care and improved clinician knowledge and understanding of available service pathways for their patients [5]. In systems characterized by long wait times and convoluted pathways to care, navigation is a timely and important innovation. Clients (and, depending on the service, their caregivers) contacting a navigation service receive a thorough needs assessment of the client’s medical and social history and the client’s and caregiver’s goals. Navigators develop meaningful relationships with clients, engaging with them throughout the care process and providing individualized resource options specific to the difficulties the client and family are experiencing, their preferences, as well as their goals. Navigators can also provide psychoeducation and advocacy, follow-up on referrals, and support interprofessional communication across the client’s care team. Models of care vary, with some programs focusing on lay navigation by peers, professional navigation by trained clinicians, or both [25]. Programs may offer short-term navigation, while others may stay with the client and/or caregiver for as long as necessary to ensure they are connected to the right supports [12]. Navigation delivery methods differ, with a mix of in-person, phone, email, and text message-based services [25]. Despite the recognized potential of navigation services to support clients and their families and the range of services available, there previously had not been any coordinated academic or clinical event that supported dissemination of learnings regarding navigation in the Canadian context. In light of the considerable uptake of navigation services across Canada, it is important that conversations regarding lessons learned, best practices, service innovations, and novel research findings take place early and often to ensure that navigation services themselves are evidence-informed and well-integrated across the country.

## Conference co-hosts: the Family Navigation Project at Sunnybrook and NaviCare/SoinsNavi at the University of New Brunswick

The *Canadian Healthcare Navigation Conference* was a joint venture between the Family Navigation Project (FNP) and NaviCare/SoinsNavi. The FNP at Sunnybrook Health Sciences Centre in Toronto, Ontario was created to support access to and transition through treatment systems for youth with mental health and/or addictions concerns and their families [10]. The FNP is a non-profit, free of charge service that was developed by families, for families. As the FNP is situated in a large academic health sciences centre, the group has a commitment to research and innovation in the field. Navigators engage with families one-on-one throughout the care process; provide expert individualized resource options specific to the difficulties the youth and family are experiencing and their goals; facilitate and follow up on connections; and negotiate challenging situations as they arise. Through this innovative model, the FNP untangles the web of the MHA system and supports youth and families in accessing timely and appropriate supports. Launched in 2017, NaviCare/SoinsNavi was a patient navigation centre in New Brunswick housed under the Centre for Research in Integrated Care (CRIC) at the University of New Brunswick (UNB) Saint John. This centre supported children and youth with healthcare needs, their families, and the care team. The research-based navigation centre facilitated convenient and integrated care to support the physical, mental, emotional, social, cultural, and spiritual needs of children/youth up to the age of 25 and their families. NaviCare/SoinsNavi employed one bilingual patient navigator who worked with clients to formulate and prioritize goals based on their unmet needs. NaviCare/SoinsNavi was the first navigation centre of its kind in New Brunswick. After four-and-a-half-years of operation, the NaviCare/SoinsNavi research project has come to a close. The team of dedicated individuals are looking forward to incorporating lessons learned by building navigation models for other populations, such as those with dementia and their care partners. As two established navigation programs in Canada, FNP and NaviCare/SoinsNavi were poised to lead and facilitate the national discussion on navigation services and ways to best support clients and their families. FNP’s and NaviCare/SoinsNavi’s clinical and research expertise as well as their existing networks helped ensure the design of an event that addressed the range of client and family needs; geographical and social contexts; and service modalities that already exist or are emerging across Canada. CRIC, the research centre that led NaviCare/SoinsNavi, will co-host the Canadian Healthcare Navigation Conference going forward, along with the FNP.

## The Canadian Healthcare Navigation Conference

In recognizing the timeliness and importance of creating a national dialogue around navigation services, the inaugural *Canadian Healthcare Navigation Conference* (https://chnconference.ca) was developed. The goal of the conference was to share existing navigation models across Canada, as well as best practices in program implementation and evaluation and ongoing research in the field of navigation. The event was planned to bring together people with lived experience, navigation service providers, trainees, researchers, and decision makers, and other relevant stakeholders who are involved with or interested in learning more about navigation services. The conference was designed to connect stakeholders for information-sharing pertaining to key lessons learned in developing, implementing, executing, sustaining, and evaluating navigation services in this realm. This inaugural conference was the first of its kind in Canada and served as an important step to ultimately create a shared dialogue around navigation services in the Canadian healthcare system. The inaugural event was originally scheduled for April 30–May 1, 2020, to be held in-person in Toronto. Due to the global pandemic, the event was rescheduled for April 15–16, 2021 and was held virtually.

### Abstract review

Abstracts were originally accepted until mid-January 2020. With the postponement of the originally scheduled conference, accepted presenters were notified that their presentations could carry over and were asked to confirm their participation once the event was rescheduled. A supplementary call for abstracts was then launched the following year, with abstracts accepted until mid-February, 2021. To ensure that the conference reflected a collaborative process and brought all voices to the table, the abstract review committee included researchers, clinicians, as well as individuals with lived experience from the Family Advisory Councils of the FNP and of NaviCare/SoinsNavi in both rounds of review. Participants with lived experience were provided honoraria for their time. Abstracts were assessed for quality based on the description of the proposed presentation (i.e., introduction/background, design/details of presentation, results/lessons learned, and conclusion/implications), relevance to the conference, and overall reviewer impressions.

### Conference sessions

The event took place over two consecutive afternoons on April 15–16, 2021, recognizing the shift to virtual format precluded full days of sessions. On day one, the first keynote presentation was delivered by Ms. Keli Anderson, President and CEO of FamilySmart in British Columbia, a peer-led mental health navigation program. Ms. Anderson is an individual with caregiving lived experience who participates in advocacy in Canadian healthcare. The second keynote presentation led the second day of the conference, and was delivered by Dr. Karen Freund, Chair of the Tufts University School of Medicine and Physician-in-Chief at Tufts Medical Centre. Dr. Freund is recognized across the US and Canada for developing models for patient navigation and care coordination in cancer among vulnerable populations. Concurrent sessions included a total of 36 presentations in key theme areas (i.e., navigation in practice, navigation in research, navigation in education, navigation in administration, navigation in policy) held across the two afternoons of the conference. Event participants were able to attend their choice of sessions via live stream and watch recordings following the sessions. Research posters (33 in total) were available for viewing in advance of and throughout the event, with a dedicated poster session held at the end of the first day that provided time for poster presenters and attendees to engage with each other and discuss their work. A panel discussion rounded out the second day of the event, focusing on the current state of healthcare navigation and future directions for the field. Panelists included Dr. Shelley Doucet (Jarislowsky Chair in Interprofessional Patient-Centred Care, Professor, and Director of the Centre for Research in Integrated Care at the University of New Brunswick), Dr. Anthony Levitt (Medical Director of the FNP, Chief of Brain Sciences at Sunnybrook Health Sciences Centre, and Professor in the Department of Psychiatry at the University of Toronto), Michele Sparling (mental health advocate, peer support champion & founder of Just Be You, member of boards and advisory committees in the mental health system) and Mary Beth Wighton (dementia advocate and author, Co-Chair and Co-Founder of Dementia Advocacy Canada).

### Registration support for attendees with lived experience

Registration rates varied by participant type (e.g., individual with lived experience, service provider, student/trainee, researcher, and decision maker), with students/trainees and individuals with lived experience offered the most reduced rates. To enable the participation of individuals with lived experience in the conference, registration bursaries covering the full registration fee were offered for individuals with lived experience. A total of 14 bursaries were provided through this program.

### Conference app

The Canadian Healthcare Navigation Conference was hosted virtually, through the Whova application. This application provided a platform on which attendees could build an individual profile and access all conference materials and sessions, including the full event agenda, keynote sessions, concurrent oral presentation sessions, poster presentations, and event sponsor exhibitor pages. Within individual session sections of the app, attendees could “like” sessions or posters, chat with each other, ask questions to the speakers in the Q&A section (and upvote questions asked by other attendees, by “liking” to indicate agreement) that would be posed by session moderators to the speakers when time allowed and/or answered by speakers following presentations, and participate in polls put forth by speakers, all synchronously or asynchronously. Session materials were also made available so that attendees could view abstracts and download session slides. The Zoom application was embedded within Whova and utilized for live stream presentations. Posters could also be accompanied by a pre-recorded video of the speaker presenting the work, and many poster presenters availed themselves of this option.

Within the app, there were also dedicated sections for viewing and contacting event attendees, personal messages, community message boards, and photo sharing. These features were utilized to encourage participant engagement. For example, the community board enabled attendees to arrange virtual “meet-ups” based on shared interests, share articles and resources, and post discussion topics. The photo feature was utilized for a photo sharing contest, where attendees were encouraged to post photos of their “work (from home) heroes,” such as children, pets, coworkers, or even ways they cared for their own mental health during the pandemic (including baked goods, outdoor spaces, etc.) The winning photo was chosen at random. A leaderboard contest was held for the duration of the event, where attendees earned points for attending sessions, engaging with session features (e.g., asking questions, “liking” sessions), posting on the community board, and participating in the photo sharing contest. All app features, including recordings of presentations, remained available to attendees for 90 days following the conclusion of the conference.

### Participants and engagement

**Fig. 1:**
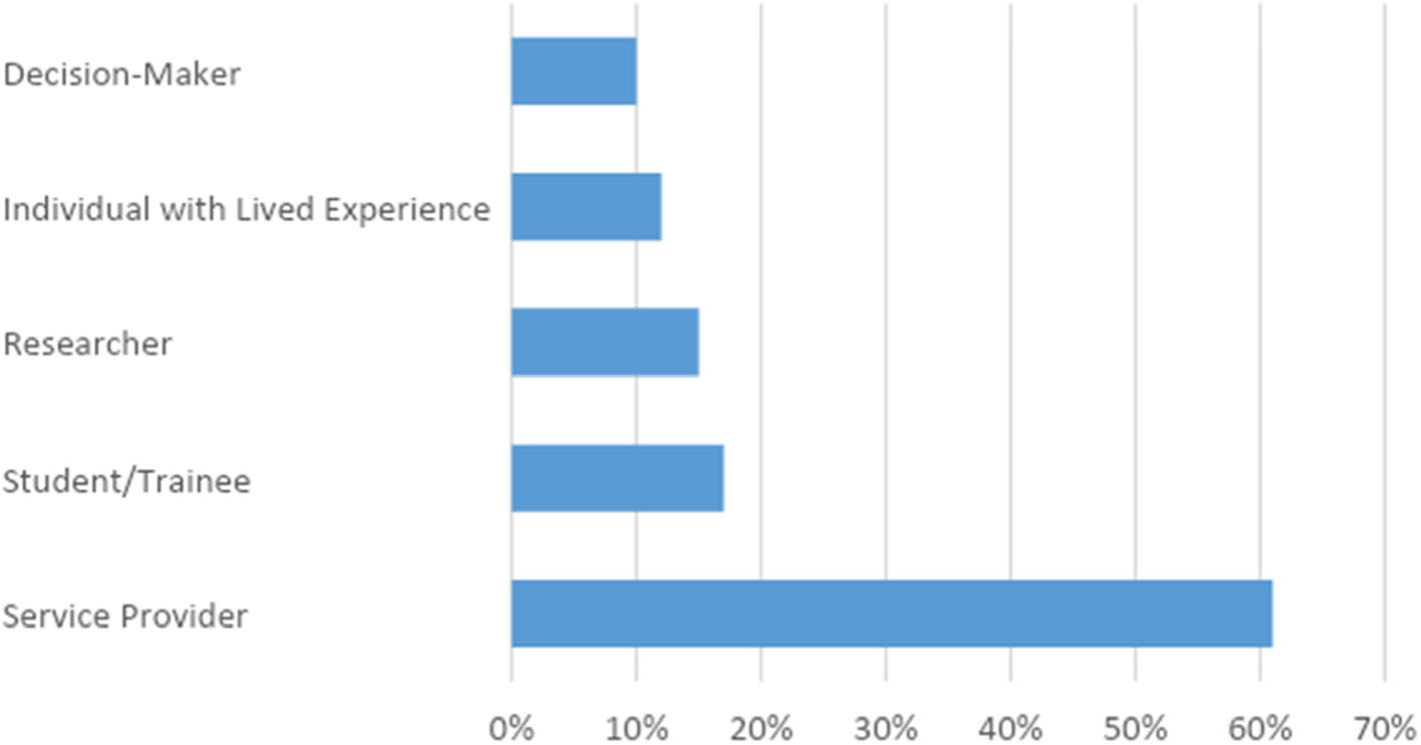
Attendee roles

A total of 200 attendees took part in the inaugural Canadian Healthcare Navigation Conference. Geographical representation included all ten provinces of Canada as well as representation from the USA, Australia, Trinidad and Tobago, and Brazil. Over 80 healthcare (public, private, and community), educational, social care, and other support organizations and agencies were represented. The majority of presenters and registrants who had planned to attend the 2020 conference re-registered for the 2021 virtual event. Attendees were able to indicate their role(s)/identification(s) upon registration, from a list of options including: individual with lived experience, service provider, student/trainee, researcher, and decision-maker. The majority of attendees identified as service providers, whereas decision-makers represented the smallest proportion of attendees (see Fig. 1). Numerous primary fields of practice/research/experience within navigation were identified, as shown in Table 1. Note that this may not be an exhaustive list, and was limited to information provided at the time of registration or in presentations; attendees may have identified with more than one of these, or may have had additional fields of interest that they did not indicate as primary in their work.

**Table 1:** Attendees’ fields of practice, research, or experience

Domain	Field
Health concern/Population served	Alzheimer’s/Dementia Autism/ASD Acquired Brain Injury Behavioural health Cancer Complex medical care needs Indigenous healthcare LGBTQ+ healthcare Mental health and addictions Multiple Sclerosis Musculoskeletal disorders Neonatal intensive care Neurodisabilities Newcomer and immigrant health Palliative care Pediatric care Reproductive healthcare Trauma care
Service setting	Academic Community Hospital Information and Referral Services Online Telehealth
Professions/Areas of practice	Medicine (including subspecialties) Nursing Public Health Rehabilitation Social Work

**Fig. 2:**
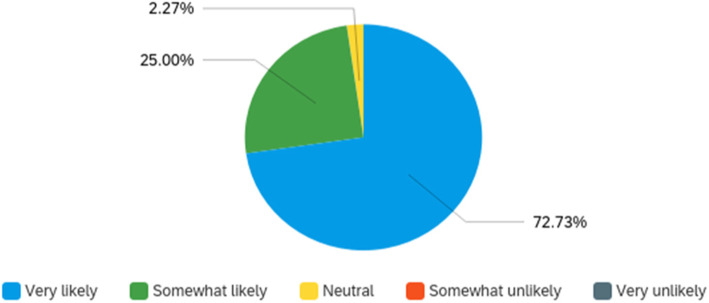
Likelihood of recommending conference

**Fig. 3:**
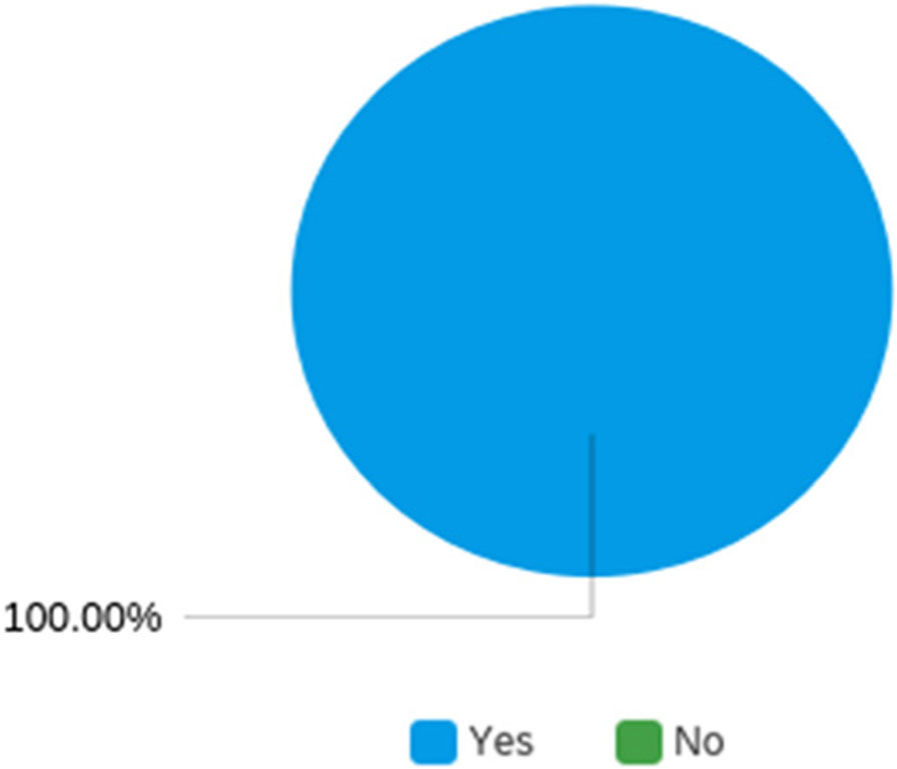
Likelihood of attending in future years

**Fig. 4:**
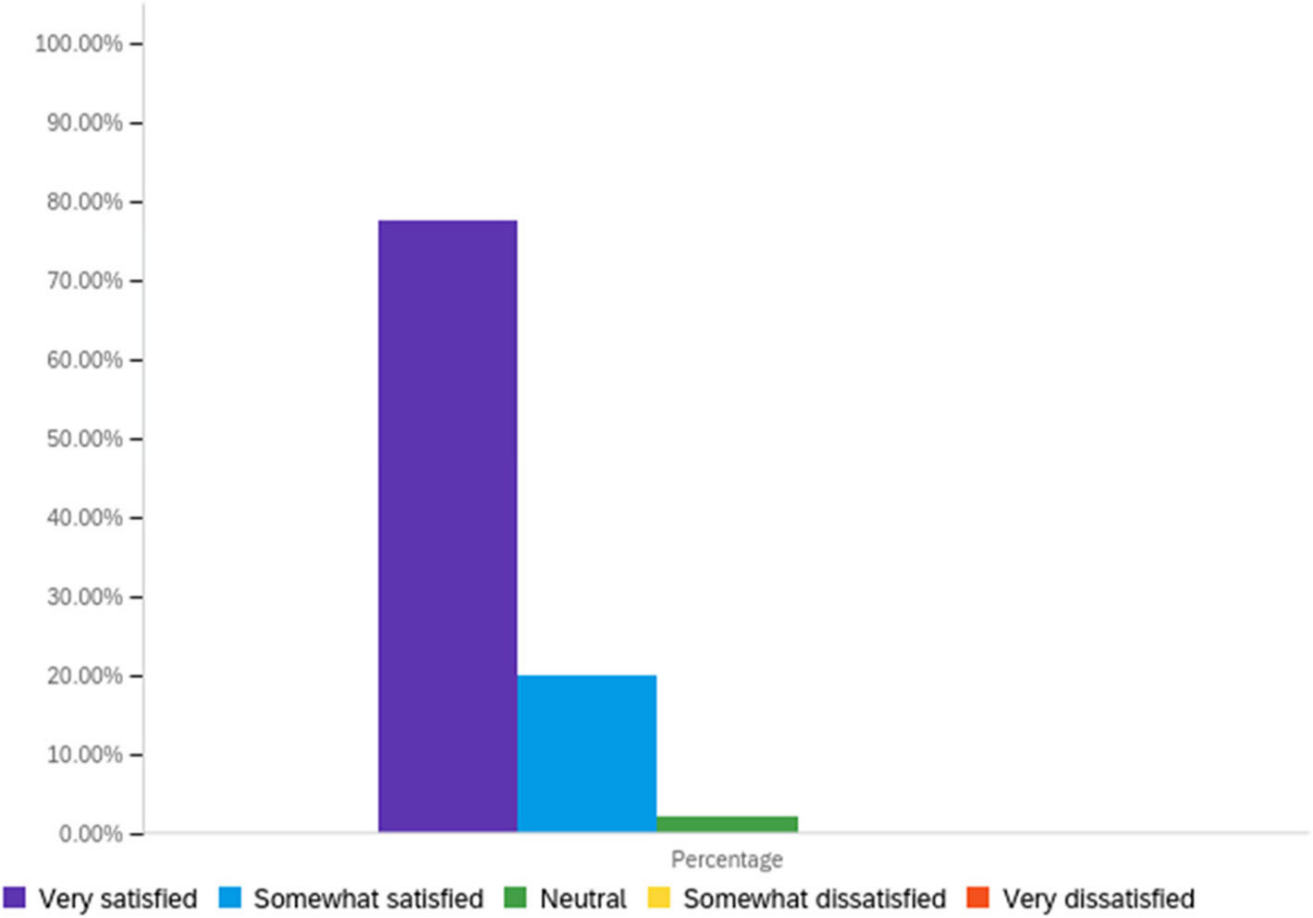
Satisfaction with the conference

**Fig. 5:**
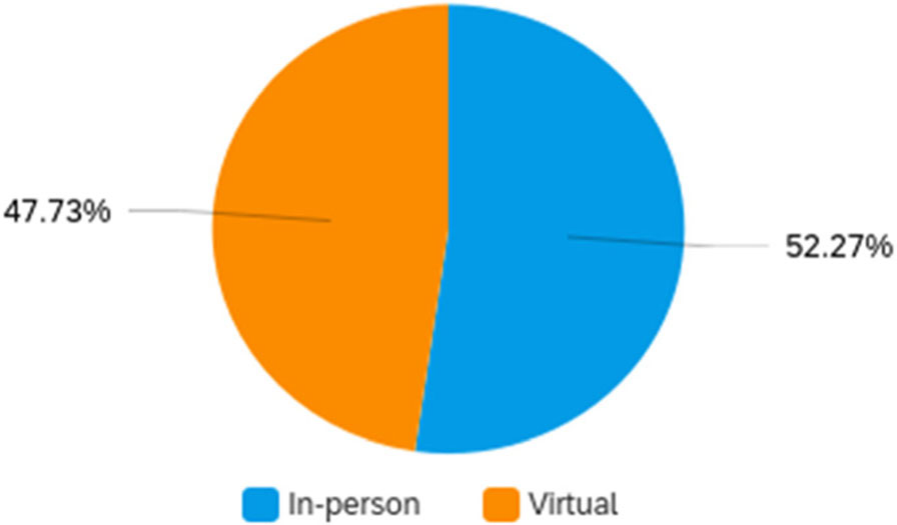
Conference format preference in future years

Attendee engagement in the virtual format was supported by the conference app, as described above. In all, there were over 60 discussion topics posted and over 800 messages exchanged on the community message boards. Over 30 photos were shared by attendees for the photo sharing contest, with over 300 likes. There were over 500 one-on-one private message interactions between attendees, indicating substantial networking opportunities. Finally, attendees “liked” sessions 228 times. Conference feedback through a post-event survey was extremely positive, which is depicted in Figs. 2, 3 and 4. Attendees also provided constructive open-text feedback that will be taken into consideration in future years, including: the desire for longer presentations during the concurrent sessions, which in the current year, had followed an academic 15-min format; a greater representation of practice-oriented presentations, which in the current year, had been equally balanced with other conference streams but may not have resonated fully with the audience due to the majority representation of service providers among attendees; and more networking opportunities, which the organizers hope to facilitate in future years through in-person or hybrid in-person/virtual conference formats. Open-text responses in the conference survey indicated that attendees most appreciated: the opportunity to connect with navigators across the country and come together as a field; the variety and quality of topics and speakers, including the engagement of the audience by the speakers; the inclusion and encouragement of the lived experience aspect of navigation; and opportunities for networking and engagement despite the virtual format. Although contradictory, this final point was highlighted by attendees as something that was done well by the conference and as an area of improvement for the conference. This may reflect the varying degree of comfort with and acceptability of connecting through technology, as seen in many areas of life and work during the COVID-19 pandemic and also evidenced by the near 50–50 divide in preference for an in-person or virtual meeting in the future (Fig. 5).

## Canadian healthcare navigation conference proceedings

### Keynotes

**Keynote 1. Ms. Keli Anderson. “There is no right path. Just the right people you meet along the way.”** The first day of the conference began with a brief welcome and opening words, followed by a keynote address by Ms. Keli Anderson. Ms. Anderson shared her experiences in finding care for her young son, including multiple occasions where she was made to feel blamed for her son’s concerns or dismissed by professionals, and how those experiences drove her to begin an organization called FamilySmart™. She emphasized that her presentation was rooted in her experience as a mother, not a clinician, researcher, or academic. When describing the navigation supports offered by the Parents in Residence and Youth in Residence at FamilySmart™, she explained that these individuals have been through services themselves, and that who people are is as important as what they are, in terms of navigator background and training. She discussed the importance of recognizing that navigating is not a straight line, as well as the importance of navigating other emotions and needs that arise when seeking care, including: shame, blame, stigma, people, relationships, health literacy, services, systems, fear, loss, and life. She also explained that FamilySmart™ Parents and Youth in Residence focus on advocating for youth and families; they do not judge or blame, do not take sides, and do not speak “for” youth and families.

Attendees thanked Ms. Anderson for an “inspirational” and “powerful” talk, and appreciated the importance of lived experience in system supports and advocacy. There was considerable discussion of navigator background in the session chat and Question & Answer section, including the differences between professional, lay, and peer navigators. When asked whether there might be specific settings or contexts where a lay or professional navigator would be better suited, Ms. Anderson responded that it can be both; that individual characteristics matter more, and that she did not believe there is a setting where professional credentials were compulsory. Also discussed were barriers for marginalized and vulnerable populations. One question posed to Ms. Anderson specifically focused on reaching rural and remote communities, and she described the importance of understanding local cultural contexts as well as partnering with services where families are already comfortable and attending in their local communities. Finally, Ms. Anderson was asked about the emotional support available to the peers in the Parent in Residence and Youth in Residence roles. She described FamilySmart™’s robust program of support, management, supervision, and ongoing clinical support, as well as the importance of directing financial resources to maintaining this care and support to ensure the wellness of peers and, by extension, the viability of the program itself.

**Keynote 2. Dr. Karen Freund. “Sustaining a patient navigation program.”** The second day of the conference also began with a keynote address, delivered by Dr. Karen Freund. Dr. Freund shared her learnings from decades of research in cancer care navigation. She highlighted that patient navigation is a system of care, not simply a person in a navigation role, and that navigation is indicated throughout the continuum of care. She described the importance of proactive anticipation of client needs and delivering support in a culturally competent manner. She identified the goals of navigation in cancer care as increasing adherence to and timeliness of care, decreasing mortality, and improving quality of life. Dr. Freund also described the care coordination systems model, which involves identifying patients in need of navigation; assessing needs and barriers; developing a plan to address barriers; and tracking and following through to completion of care. She explained that the tasks of patient navigators are to translate, into lay language, what clients can expect in their care; promote understanding of the healthcare system; help ensure clients are able to attend their appointments; coach and provide emotional support; assist with addressing barriers to care; and track clients through to completion of care. She indicated that a unique aspect of patient navigation is addressing clinical care needs in the context of clients’ own social needs. Finally, she advised that organizations wishing to evaluate their outcomes first identify what is important to the organization and its stakeholders, and design metrics that follow.

Attendees were enlightened by Dr. Freund’s informative talk. They discussed different modes of delivering navigation supports, such as face-to-face or by phone, with agreement that the focus should not be on the modality, but whether and how good rapport could be established. Stakeholder input was recognized as integral in identifying the need for navigation programs and the supports they can provide in response to local contexts. There was also discussion of how to make navigation more integrated and coordinated, and the value of team approaches over navigating in isolation. The importance of making care accessible for clients was also described, for example, by having navigators attend clinical sites, introduce themselves to patients, and offer navigation supports. Furthermore, the role that navigators can play in addressing social needs, alongside or instead of physicians and other community health workers, was also explored. Finally, the need for greater understanding of outcomes of navigation was addressed. For example, mortality was identified as an important downstream outcome for investigation, yet Dr. Freund indicated that deciphering the impact of navigation specifically on this outcome is difficult, even in cancer care where such outcomes are carefully tracked.

### Concurrent and poster sessions

Following the Keynote addresses on both days of the conference, oral presentations took place in Concurrent Sessions. The poster session took place at the end of the first day of the conference. These sessions focused on four themes: Navigation in Practice, Navigation in Research, Navigation in Policy and Administration, and Navigation in Education.

Oral and poster presentations covered a range of topics, including: reporting research findings on navigator roles and navigation nomenclature in various systems/fields; literature reviews; research and evaluation outcomes of navigation supports; theoretical considerations in navigation approaches; developing navigator training; development and features of navigation programs; and family caregiver experiences and needs. The full list of concurrent and poster presentation titles and authors is available on the conference website: https://chnconference.ca. Attendees and presenters engaged with each other extensively. Across all session topics, there were common discussions that emerged pertaining to approaches to navigation; roles of navigators; evaluation and quality improvement; the involvement of lived experience in navigation; and the effects of the COVID-19 pandemic on navigation supports.

#### Approaches to navigation

A significant proportion of session presentations and discussions centred on approaches to navigation supports. While numerous presenters and attendees noted the lack of standardization in the field of navigation, particularly in terms of modes of program delivery, naming of navigator roles, and program features, many commonalities and innovative approaches emerged through presentations and participant discussions. This was evidenced through presenters’ discussions of the need for navigation support, navigation program models and goals, features of effective navigation support, technology, and challenges and opportunities encountered.

##### Need for navigation support

The need for navigation was attributed to poorly integrated, convoluted, and siloed service systems with varying points of access, long wait times, and bureaucratic regulations that were difficult for clients and families to understand. Navigators were viewed as having the ability to support clients facing long wait times for services, experiencing difficult transitions between services, and those that are falling through cracks in the system due to the level of need/complexity experienced being too high or low for available services. Social determinants such as culture, race, language, and other factors that can create and exacerbate health inequities were discussed as factors that navigators can strive to understand and address (e.g., by understanding the needs of marginalized and vulnerable groups, for example, by providing extended support for newcomer groups).

Also described were the demographics, care needs, and risk factors of clients. In this vein, presenters discussed how children and youth can face a lack of continuity of care if providers are not able to suggest follow-up treatment after a hospital stay. On the other end of the lifespan, medical advances mean that clients are living longer and the disease burden is changing, suggesting pressures on the healthcare system and related services will continue to grow. Furthermore, complex care needs requiring an array of supports frequently have no services that integrate sectors and systems. Such circumstances leave clients and caregivers spending significant time trying to find the services they need for themselves or their loved ones, particularly if they do not know how to begin or do not know what supports might be available. Connecting them with navigators was described as a way to alleviate this burden and potentially alleviate healthcare system spending.

##### Navigation program models and goals

Many presenters shared navigation programs and supports offered, including, but not limited to: a wellness navigation component of a community health model that can support any condition; navigation for individuals with multiple sclerosis; navigation for pregnant women at high risk of having their infants apprehended at birth; navigation for families of children with neurodevelopmental disabilities; navigation for newcomers; navigation for individuals (including children and youth) with mental health and/or addictions concerns and their families; navigation for behavioural support needs; navigation for Indigenous individuals; navigation for dementia patients and their caregivers; and navigation for individuals (including children and youth) with complex medical care needs and their families.

Presenters discussed models of care applied in navigation supports, such as patient-centred, family-centred, and person-centred care, as well as relationship-based care as a means of building on all three. A family resilience theoretical framework was also seen as aligning well with navigation work. Culturally-informed models were described as a means of bridging, linking, and mediating between groups with different cultural backgrounds, while creating awareness, understanding, and respect of traditional beliefs and values. Across models of care and support needs addressed through navigation, presenters emphasized the importance of a collaborative team approach that bridges providers and systems, recognizing in particular the siloed nature of healthcare and other systems and the ability of navigators to address this fragmentation. This was surmised to result in improved health outcomes and reduced health disparities, more efficient systems, and increased capacity across systems.

Numerous goals of the various navigation programs described were also addressed. Goals focused on the client included ensuring the client is making informed decisions or has the information necessary to make decisions regarding their care; ensuring care is developmentally appropriate; alleviating barriers to care, etc. Goals focused on the family included involving families in the client’s care; reducing family stress; and developing relationships with caregivers. Goals relating to providing varied supports were described, including developing public educational tools and combining navigation and clinical service. Goals related to supporting access to and transitions in care were detailed, such as improving access to community resources and supports; bridging clients from hospital and primary care to community-based supports; supporting aging in place and end-of-life care at home; ensuring clients and families have been connected to and retained in a recommended service; and reducing hospital recidivism. Many navigation programs also had goals related to developing system capacity, for example, by growing their networks of resources that can be offered to clients, creating linkages across the system, and supporting integration of navigation with service delivery. Presenters also described the importance of goals associated with the sustainability of navigation supports (e.g., through continued ability to ensure relevance and effectiveness, demonstrate impact, and secure funding) to ensure navigation supports remain available to those in need.

##### Features of effective navigation support

Although presenters noted a lack of standardized approaches across navigation programs, they also shared elements of effective supports for clients. These elements included the importance of embedding navigation within systems of care. Navigation can thereby be an open door rather than turning away clients in need, and then help guide clients to the “right” door, as well as “softly” discharge clients upon the conclusion of navigation service so that they are welcome back to receive additional navigation support whenever needed. Another element involved anticipating clients’ needs and proactively linking clients and families to needed supports, such that the role of the navigator will progress along with the client’s care trajectory (e.g., in progressive illnesses, such as dementia, or in cases with evolving needs, such as palliative care).

Presenters also highlighted the importance of effective communication at multiple levels, that is, with clients and families and with providers and decision-makers across the appropriate health and social care systems. Working collaboratively with these groups and sharing resources when possible was seen as a way to enhance care for clients and families. Also emphasized was the importance of ensuring services are recovery- and trauma-informed, ensuring connection and rapport with clients, and facilitating informed choice in care. Being flexible and creative were acknowledged as key when providing navigation supports. For example, a number of navigation programs described providing mobile supports to more effectively address their clients’ needs.

The importance of understanding local contexts and needs was acknowledged, for example, by offering multi-lingual services; integrating biopsychosocial and spiritual approaches; developing trust within Indigenous communities; and understanding and appreciating the worldviews of marginalized communities. The importance of learning about and listening to local communities, being respectful, working to understand the community’s values, and getting in touch with key contacts in the community were all emphasized as ways to provide navigation supports that would be responsive to community needs; integrate effectively with local cultures and contexts; and ensure navigation supports are ultimately accessible for those in need. Similarly, interprofessional and intersectoral collaboration was seen as key to success by supporting capacity-building and system transformation. For example, navigators and navigation programs were seen as able to build system capacity by sharing knowledge and resources and creating educational products. Underscoring these discussions were funding considerations, including how to keep programs running and resource information live and current in the face of cyclic and discontinuous funding.

##### Technology

Technology considerations were discussed, such as the use of electronic records; web-based supports; in-person supports; phone and email supports; providing e-learning resources for providers; app-based information and resource-sharing; and ultimately, ensuring that services are accessible for clients and families in the format offered. The need for infrastructure support, data governance, customizability, and collaboration for information-sharing was noted. Nevertheless, technology was recognized as a powerful adjunct and facilitator for navigation services.

##### Challenges and opportunities

Finally, many presenters described challenges and opportunities encountered in their navigation work. Lack of awareness and understanding of navigation on the part of clients, families, and system providers often limited the ability of navigators to effectively support clients, which many associated with the need for standardization of the navigator role. Professional boundaries also presented difficulty at times, in that navigators would sometimes be unable to provide supports that clients desired, but were out of scope for the navigator role (e.g., direct psychotherapy). Maintaining an up-to-date repertoire of services to which clients could potentially be connected was another challenge noted, as information fluctuated regularly. Finally, funding was a challenge discussed by many presenters. For example, some organizations were volunteer-driven, some operated based on being able to obtain consecutive grants or philanthropic support, and many were seeking sustainable funding.

Presenters also acknowledged the potential for navigation to be seamlessly integrated into healthcare and other systems. Navigators could reduce workload for care providers by supporting client-provider communication and helping to develop and apply established referral and transition pathways with warm handoffs, ultimately enhancing clients’ and families’ care experiences. However, for such integration to be effective, there was a recognized need for commitment from organizational senior leadership teams and local governments, as well as a need for local partnerships (e.g., hospital-community partnerships for continuity of care; educational-clinical partnerships for student placements). Working collaboratively was seen as a means to decrease duplication of navigation programming efforts and improve sustainability.

#### Navigator roles

The benefits of designated and focused navigation roles were discussed, including the ability for navigators to be able to direct their energy and resources on navigation activities, developing system knowledge, and facilitating system coordination and management. Furthermore, although navigation falls within the scope of many professional roles, there was agreement that navigation works best as a standalone role. Navigators can save time for other professionals to focus on direct care by committing to thorough navigation and can even bring care teams together to advance the care plan when it is otherwise stalled. This was also seen as a means of decreasing duplication of supports across systems and could thereby improve clients’ care trajectories.

With regard to navigator roles, there was also considerable discussion across conference sessions of professional and lay navigators, as well as peer navigators. Professional navigators were viewed as those with clinical educational background and training (e.g., nurses, social workers, etc.), while lay navigators were noted to not have a professional designation, but may complete relevant in-service training and professional development opportunities. Peer navigators were described as those with lived experience of the care needs being navigated for, would have been through the systems being navigated, and would therefore be able to provide support through the lens of someone with experiential knowledge. Peer navigators were typically considered lay navigators, although there was acknowledgement that peers may often have clinical educational backgrounds and therefore be considered professional navigators as well. There was also acknowledgement that blended models with lay and professional roles working in tandem could be beneficial as long as the purposes and goals of the program are met and scope of practice is maintained.

A wide range of role titles were identified for people doing navigation (e.g., patient navigator, family navigator, wellness navigator, community navigator, care advisor), along with a need to understand the functional distinctions of the various roles and devise common nomenclature. Similarly, a wide range of functions were recognized for individuals who identified as navigators (e.g., care transitions, access to diagnosis, psychosocial support, care coordination, etc.), which highlighted a possible need for regulation of the navigator role or standardized training (with appropriate stakeholder consultation in the development processes). This was thought to be a way to limit confusion, especially for clients, and support communication with other care providers. Navigator roles were also contrasted with other roles that exist in different care settings, such as case managers. Much discussion centred on the importance of continuing to explore these navigator role meanings and purposes as well as to consistently define these roles. Also highlighted was consideration of standardization and certification of these roles to support clear scope of practice for navigators and to ensure these roles are clear to clients of navigation services.

Also discussed was the importance of building navigation staff capacity, which included developing knowledge (e.g., understanding of the healthcare and other systems, community resources, information about mental health, information about other chronic illnesses), and skills (e.g., professional boundaries, record management, motivational interviewing), in relation to client needs, and ensuring training opportunities were repeated at regular intervals so that the navigator could maintain relevant and current training. Finally, when training navigators, there was indication of the need to demonstrate patient-centredness through learner-centred approaches (e.g., embracing difference, meeting students where they are); supporting creativity; providing practical experience through opportunities to find and consolidate information and resources; and opportunities to develop knowledge of various intersecting systems to most effectively and holistically support clients.

#### Evaluation and quality improvement

The importance and use of program metrics was highlighted. Evaluation was acknowledged to be of importance to understand what elements of navigation programs and processes work as anticipated and how outcomes transpire for clients and families. For example, numerous presenters described adjusting their work in response to findings from quality assurance and quality improvement activities, while others also monitor administrative data (e.g., hospital admissions and emergency department visits). Attendees also indicated that they were desirous of support with finding and developing appropriate evaluation measures for their navigation programs.

There was discussion of experiences in undertaking evaluation development and planning work and how tools and measures were developed and selected through a stakeholder- and evidence-informed process. Presenters also emphasized the importance of embedding client voices into the evaluation of programs and engaging all stakeholders in evaluation activities. Presenters acknowledged the importance of measurement and evaluation not only to inform system planning and support program sustainability, but also to move the field of navigation forward.

Presenters and attendees also discussed the evidence base around navigation. Client satisfaction with navigation was discussed, pointing to appreciation for the practical linkages and supports from navigators that go beyond list-giving, appreciation for continued relationships, and valuing navigators’ abilities to clarify opaque systems for clients. There was also discussion of outcomes for referred service providers working with navigators, with some indication that providers are desirous of continued relationships and resource-sharing. Consequently, there was an identified need for greater understanding of the specific processes and approaches of navigators that help achieve positive outcomes for clients, families, and system providers. Many noted the lack of evidence and literature on longitudinal and health outcomes of navigation, and hoped that the Canadian Healthcare Navigation Conference can shed light on this over the years.

#### Lived experience in navigation

Evident in nearly all presentations was the inherent necessity of lived experience to guide navigation services. In many cases, navigation programs had begun through the advocacy efforts of clients and caregivers, grassroots patient- and family-led endeavours, or through community consultations that revealed public appetite for navigation supports. Many navigation programs were thus driven by and co-designed with individuals with lived experience to address the needs as experienced by clients and families traversing systems of care.

People with lived experience were able to identify necessary program elements that would not have been immediately apparent to those who had not been through these systems themselves, such as a preference for phone and email navigation supports rather than in-person supports. From the perspective of individuals with lived experience, the value of navigation was its potential to alleviate the stress and burden associated with navigating care for themselves or their loved ones by providing information and appropriate resource options and connecting clients and families to supports co-designed by people who have “been there,” thereby helping them feel connected to someone who understands and is “in the boat” with them. Lived experience was also highlighted as a teaching tool that could enhance the learning of students training in navigation, whereby individuals with lived experience could share their experiences as a client or caregiver accessing care. Many presenters with lived experience appreciated and emphasized the important role that navigation supports can play in giving clients and families hope.

Embedding lived experience in navigation programs was described as an ongoing process that extended beyond program design stages and involved client voices in ongoing program activities even after program implementation. Presenters emphasized the importance of ensuring inclusivity, diversity, and representation when involving lived experience, to promote learnings and program activities that benefit the wide range of clients served by the program. In addition to peer navigator roles previously described, ways to involve individuals with lived experience, beyond the co-design stage, mentioned by presenters included: roles on patient and family advisory councils; hosting workshops; developing educational and resource materials; reviewing tools and information that would be presented to program clients; informing research; and guiding program strategic directions and priorities. Presenters indicated the importance of providing options and flexibility for the involvement of individuals with lived experience, for example, through standing meetings as well as ad hoc committee options that would allow advisory council members to be flexible with their time commitment.

#### COVID-19 pandemic

Underscoring nearly every presentation was mention of the COVID-19 pandemic – not only for its impact on the conference as originally planned but more importantly, for its impact on considerations for the delivery of navigation services. Presenters discussed programming that had been cancelled (e.g., in-person family events and activities), programming that had shifted to virtual formats (e.g., workshops), and programming that fortunately, already had been established in formats that were effective during the pandemic (e.g., phone- and email-based navigation supports). Many attendee questions to presenters inquired about the shifts and changes made during the COVID-19 pandemic, and presenters shared the ways in which their services were modified (e.g., shifts to virtual offerings) or their work had changed (e.g., increased difficulty connecting clients with supports due to service closures, increased numbers of clients seeking navigation supports). Presenters and attendees acknowledged the important next step of identifying and understanding how the pandemic has impacted clients’ and families’ experiences of navigation supports.

## Panel presentation: “Healthcare navigation: where are we now and where to next?”

The panel presentation rounded out conference events through a discussion of the most critical next steps in advancing navigation in Canada. Attendees shared appreciation for the panel format as a culmination of the conference events, and actively engaged with panelists for further discussion following their presentations.

Panelists described the need to explore and come to agreement on navigation as an occupation. Although navigation is performed as a function of many roles, it is emerging as a standalone function in the healthcare system, and other systems, yet risks becoming a fragmented system of support in itself. Thus, clarity is needed regarding what navigation is and what a navigator is, who is delivering navigation, to whom navigation is being directed (e.g., patient, caregiver, care team), what is being navigated (e.g., navigation for diagnostic subgroups, navigation to services within a particular institution), how the navigation is being performed (e.g., phone, email, web, educational materials), and what the level of commitment is to the navigation role specifically (e.g., full time, part time, or part of another role entirely). Also identified through further discussion was the role that navigators can play in identifying gaps in the system. Panelists emphasized that navigators are not expected to fill gaps in the system, but to assist their clients in light of the state of available services. Identified gaps can be presented to healthcare administrators and decision-makers to effect change.

Although features of navigation supports will ultimately vary in line with local context and needs, as well as program goals, panelists also highlighted regulation as a means to maintain consistency and public confidence. Identified priorities included establishing a regulatory body and creating standards of practice and a code of ethics, and by extension, developing training standards. Regulation could then enable shared knowledge and shared vision among navigation programs across the country. Consistent training may help ensure navigators are able to identify and resolve barriers to care while appreciating real and perceived barriers, thereby overcoming the barriers navigators themselves experience when finding appropriate care for their clients, enabling adherence to planned care pathways, and ultimately improving outcomes for clients, families, and systems. Further, discussion during the audience question and answer period highlighted the need to develop training opportunities that address the commonalities across navigation programs by, for example, teaching skills that can be used in a variety of contexts and with different client needs, such as motivational interviewing. With regard to ensuring quality and consistency in lay and professional navigator training, panelists emphasized that quality can be a spectrum, and should extend beyond disability rights and align with human rights frameworks. Thus, when implementing these values, training opportunities would have many commonalities, but with some divergence depending on context-specific needs and goals. Navigator training was also viewed as necessarily linked to navigation programs to be able to offer valuable practical experience to trainees. There was also caution suggested in considering regulation, with due consideration of the pros and cons of becoming a regulated profession. For example, regulation might create tension with the creativity necessary in navigation work; however, regulation might also better protect the public by ensuring a level of competence across the profession that could assure the maintained reputation of affiliated programs.

Virtual navigation was also addressed by panelists as a result of the ongoing pandemic. Virtual navigation was suggested as a means to improve service levels that were previously only provided in person, if at all, which could increase accessibility for those who could not access in-person supports. Further discussion on this topic centred on the effects of the pandemic on the stress and mental health of society, with recognition that more issues could arise in future as a result of the pandemic. Panelists acknowledged weaknesses in the Canadian healthcare system that became evident as a result of the pandemic, and urged navigation programs to remain flexible and keep track of changing services (e.g., services becoming available virtually). Caution was urged to be mindful of technology needs, in order to promote accessibility without unintended consequences associated with inadvertently excluding those who could not actively utilize virtual supports.

Also described by panelists was a need for more research on navigation, including: outcomes-level research, along with a need for agreed-upon and standardized outcomes; return-on-investment research; research on lay, professional, and blended models; research on experiences of patients and care providers; and moving from single site evaluations conducted in house to multi-centre research with external evaluators. All of these research priorities would need to engage patients, care providers, and health administrators/decision makers in all stages of the research process to ensure priorities match those of stakeholders.

In addition to engaging stakeholders in research priorities, there was a marked desire by panelists for the inclusion of the voices of underrepresented groups in all stages of navigation program planning, most notably, in the early stages of planning and when building systems of care. Attendees were urged to consider their service users and any voices that might be missing, to ensure that people are included who understand their communities and their access and service utilization barriers, which can improve cultural communication and support the empowerment of patients and families.

Finally, panelists identified the need to conceptualize navigation from a human rights perspective. Patient-centred and strengths-based models were described as extremely valuable, and a human rights framework would provide a critical lens to address discrimination, restricted social supports, built environment needs, privacy, and confidentiality. These considerations could be addressed, in part, by ensuring the voices of lived experience were actively involved in program planning. When asked about how to facilitate the inclusion of voices of lived experience, panelists suggested starting by reaching out to communities and finding out how they want to be engaged. They suggested identifying key informants, that is, those that are trusted in the community and have knowledge and insight to contribute regarding the gaps experienced by their communities. They also suggested that organizations regularly check in with themselves and consider whether there are missing voices, whether those with lived experience who are giving their time are being heard, respected, understood, and truly valued for their expertise, rather than dismissed. Attendees were also encouraged to ensure engagement efforts embrace authentic co-design, participation, and collaboration, rather than tokenism. People with lived experience specifically were encouraged to be assertive with organizations that may be speaking for them without involving them. Finally, panelists encouraged directing funding to organizations that embrace engagement to consistently promote active engagement with individuals with lived experience in navigation programming.

A poll of all attendees conducted during the panel session identified the top five critical next steps for navigation as the following:

1. Agreement on the definitions of navigation and navigator
2. Regulation and training of system navigators
3. Equity, diversity, inclusion, and accessibility in navigation
4. Creating models for integration of peer/lived experience navigation with professional navigation
5. Regional coordination of new and existing navigation services

## Future activities

The inaugural Canadian Healthcare Navigation Conference was, overall, very successful. To ensure attendees and others who were not able to attend are able to access information about the 2021 conference, the abstract book is available on the conference website. The conference organizers have developed a newsletter that will keep interested attendees and others with an interest in navigation informed of relevant news and events. Newsletter subscribers are regularly invited to submit content to share with the network. Furthermore, attendees have indicated a desire for a Navigation Community of Practice to be developed for participants to continue to share their work and lines of inquiry, and to continue to learn from each other. This is currently being explored by the Canadian Healthcare Navigation Conference organizing committee. Finally, this event is now intended to become an annual conference that brings together stakeholders involved with or interested in learning more about navigation services, so that the critical next steps in navigation identified through the conference can be addressed and so that people with lived experience, navigation service providers, trainees, researchers, and decision makers can continue to develop and share best evidence and practices in the field.

## Data Availability

n/a

## Abbreviations

CEO: Chief Executive Officer
CMHA: Canadian Mental Health Association
COVID-19: Coronavirus Disease of 2019
CRIC: Centre for Research in Integrated Care
FNP: Family Navigation Project
HIV: Human Immunodeficiency Virus
Q&A: Question and Answer
TM: Trademark
UNB: University of New Brunswick
USA: United States of America
